# Obstetrical Observations and Reflections

**Published:** 1819-02

**Authors:** W. Hamilton


### ORIGINAL COMMUNICATIONS.

*For the London Medical and Physical Journal.*

### *Obstetrical Observations and Reflections;* by W. Hamilton, M.D.

ON perusing the critical department in the 237th Number of your useful Journal, I was much pleased with your notice of the excellent report of Dr. Clarke, of the Lying-in Hospital, Dublin. His practical observations on the result of upwards of ten thousand cases of midwifery are valuable indeed, and ought to make a very deep and lasting impression on the junior practitioners of that useful art; particularly at a time like the present, when unfortunate cases are more frequent than has been observed for years.

In his remarks on ordinary cases of labour, he is persuaded that it greatly contributes both to the safety of the mother and child, to allow the uterus *gradually* to empty itself during labour; and, with a view to secure its more perfect contraction, he has been for years in the habit of pursuing the fundus uteri with *a hand on the abdomen*, till the fœtus be expelled. Such pressure also tends much to prevent profuse hæmorrhage, syncope, or retained placenta, &c. Here, in conformity of this practice, I would beg leave to call the attention of practitioners more generally to this plan; as I have also, for years, repeatedly witnessed its utility, often with the most agreeable surprise; not unfrequently delivering in a few minutes a lingering or laborious case, which had occupied the attention of the midwife for days, without any other assistance than the application of the left hand to the tumor of the abdomen (using as much pressure as the nature of the case required) during the pain, while with the right the progress of the fœtus was observed. This simple practice not only does away the use of all the different methods of many practitioners here, of using towels and bandages, of different forms, girt round the abdomen; but will, in general, be found sufficient to render the vectis and forceps almost useless: or, as Dr. Clarke justly observes, "it is so long since he has had occasion to use them in private practice, that he is persuaded a fair opportunity of doing it with advantage does not occur once in a thousand cases:" even in the hospital they were used only about once in eight hundred.

As we are taught most of those sudden and alarming deaths which succeed natural labours are attributable to the sudden collapse of the larger blood-vessels, on the removal of the abdominal pressure, I am in the habit of ordering the hand of an assistant to be kept on the body till some time after the placenta be expelled; and the patient generally expresses much satisfied with the support it affords. Of this description, I have only met with one case in nearly six hundred; and even here it was more from an adherent placenta, as the uterus never contracted after its removal:—syncope succeeding, with convulsions, indicating internal hæmorrhage, soon terminated the melancholy scene.

In another instance, recently, where the patient had a very quick and favourable labour, the same untoward symptoms succeeded a few minutes after the natural expulsion of the placenta, and threatened for several hours immediate dissolution, notwithstanding the free use of brandy, opium, ether, hartshorn, &c. &c. Still, as was evident afterwards, the abdominal pressure, and stimulating contractions of the uterine region, alone saved her; as, on the succeeding day, a mass of coagula, nearly as large as the whole volume of the child, was expelled.

The beneficial effects of this practice was rendered still more obvious by the speedy recovery of a late patient from the worst species of placental presentation, between the eighth and ninth month of pregnancy. She had been flooding profusely at intervals for three days, under the management of a very experienced midwife, who kept anxiously waiting the advancement of the child, or the appearance of pains; neither of which succeeding, and the sufferer nearly exhausted, fainting and scarcely able to articulate from the great loss, the right hand (the left being placed on the abdomen) was introduced, and with some difficulty passed through the centre of the placenta, ruptured the membranes, found the child even above the projection of the spine, brought down the feet, and delivered her, at the interval of three or four pains, of a fine living male child. Continuing the hand on her body, supporting and contracting it occasionally, the after-birth soon followed; and it was remarked that neither syncope nor loss of blood succeeded; and her subsequent recovery was as quick as from her former confinements.

As it is equally evident that the pressure and support here recommended are as proper, as it is clear that the general cause of these sudden deaths is want of contraction, and consequent internal hæmorrhage, it is unnecessary for me to add farther examples. I shall, therefore, conclude for the present, by begging leave to recommend the subject to the serious attention of your practical readers, in hopes this dangerous description of cases may become as rare as the puerperal peritonitis is now rendered, by the exhibition of a few large doses of calomel, as formerly recommended.

*Ipswich; Nov.* 12, 1818.

