# Corrigenda

**Published:** 1819-01

**Authors:** 


					? 121, ? 3, for W. Hamilton, M.D. read fV, H. Surgeon.

				

